# A novel PIP_2_ binding of εPKC and its contribution to the neurite induction ability^1^

**DOI:** 10.1111/j.1471-4159.2007.04702.x

**Published:** 2007-09

**Authors:** Yasuhito Shirai, Takuya Murakami, Maho Kuramasu, Leo Iijima, Naoaki Saito

**Affiliations:** Laboratory of Molecular Pharmacology, Biosignal Research center Kobe, Japan

**Keywords:** actin, neurite outgrowth, neuroblastoma, phosphatidylinositol 4,5-bisphosphate, protein kinase C

## Abstract

Protein kinase C-ε (εPKC) induces neurite outgrowth in neuroblastoma cells but molecular mechanism of the εPKC-induced neurite outgrowth is not fully understood. Therefore, we investigated the ability of phosphatidylinositol 4,5-bisphosphate (PIP_2_) binding of εPKC and its correlation with the neurite extension. We found that full length εPKC bound to PIP_2_ in a 12-*ο*-tetradecanoylphorbol-13-acetate dependent manner, while the regulatory domain of εPKC (εRD) bound to PIP_2_ without any stimulation. To identify the PIP_2_ binding region, we made mutants lacking several regions from εRD, and examined their PIP_2_ binding activity. The mutants lacking variable region 1 (V1) bound to PIP_2_ stronger than intact εRD, while the mutants lacking pseudo-substrate or common region 1 (C1) lost the binding. The PIP_2_ binding ability of the V3-deleted mutant was weakened. Those PIP_2_ bindings of εPKC, εRD and the mutants well correlated to their neurite induction ability. In addition, a chimera of pleckstrin homology domain of phospholipase Cδ and the V3 region of εPKC revealed that PIP_2_ binding domain and the V3 region are sufficient for the neurite induction, and a first 16 amino acids in the V3 region was important for neurite extension. In conclusion, εPKC directly binds to PIP_2_ mainly through pseudo-substrate and common region 1, contributing to the neurite induction activity.

Protein kinase C (PKC) plays pivotal roles in proliferation, differentiation, and apoptosis etc. The PKC family consists of at least 10 subtypes that are classified into three groups based on the structure of their regulatory domain (RD) (Nishizuka 1992; Shirai and Saito 2002; Newton 2006). Conventional PKCs (α, β1, β2, and γ) have two common regions, C1 domain and C2 domain, in the RD. The former is responsible for diacylglycerol (DAG) and phorbol ester binding, the latter binds to calcium. Thus, calcium and DAG are required for the activation of conventional PKCs. On the other hand, novel PKCs (ε, δ, η, and θ) are activated by DAG, but not by Ca^2+^ because novel PKCs lack the C2 domain. Atypical PKCs (ζ and λ/ι) are insensitive to both Ca^2+^ and DAG because of lack of the C2 domain and one of the C1 domains. Each subtype shows different enzymatic properties and distinct tissue and cellular distribution, suggesting specific functions of each PKC subtype (Ohno 1997), but the individual functions have not been fully understood.

Among them, εPKC is abundant in the central nervous system and is thought to play important roles in nervous system (Tanaka and Nishizuka 1994; Akita 2002). Specifically, εPKC is localized at nerve terminus and seems to mediate synaptic function (Saito *et al.* 1993; Prekeris *et al.* 1996). Indeed, εPKC induces neurites outgrowth in during neural differentiation with various stimulations (Hundle *et al.* 1995; Zeidman *et al.* 1999). For the neurite-induction ability, the actin-binding site (ABS) between the first half of C1 domain (C1A) and second half of C1 domain (C1B) is important (Prekeris *et al.* 1996, 1998;Zeidman *et al.* 2002). Interestingly, it has been shown that the neurite-induction ability of εPKC is independent of its catalytic activity; regulatory domain of εPKC (εRD) also induces neurite outgrowth (Zeidman *et al.* 1999). However, in the case of the εRD-mediated neurite induction, deletion of ABS is not effective; RD of an ABS-deleted mutant still had strong neurite induction ability, while full length of the ABS-deleted mutant had about 50% less activity (Zeidman *et al.* 2002). These results suggest that there is an additional factor, in addition to actin binding, important for the neurite induction by εPKC, especially by εRD.

Phosphatidylinositol 4,5-bisphosphate (PIP_2_) is membrane phospholipids. It is well established that PIP_2_ is involved in the regulation of the action through regulating actin-binding proteins (Toker 1998). To date, many actin-binding proteins which can bind to PIP_2_ have been reported (Toker 1998; Gorbatyuk *et al.* 2006). In addition, phospholipids including PIP_2_ can activate some PKC including εPKC (Toker *et al.* 1994) and direct interactions of phospholipids to some PKCs have been reported (Huang and Huang 1991; Sanchez-Bautistra *et al.* 2006). However, there is no direct evidence that εPKC binds to PIP_2_. We, therefore, investigated the possibility of the direct binding of εPKC to PIP_2_ and its correlation with the neurite outgrowth.

## Materials and methods

### Materials

SH-SY5Y cells were purchased and fetal bovine serum was obtained from Riken cell bank (Tokyo, Japan) and Sigma (St Louis, MO, USA), respectively. FuGENE 6 transfection reagent (Roche Molecular Biochemicals, Indianapolis, IN, USA) and PIP_2_ were obtained from Roche Molecular Biochemicals. Rabbit anti-green fluorescent protein (GFP) antibody was produced by us. GFP–fused pleckstrin homology domain (PHD) of phospholipase Cδ (PLCδ), named GFP–PHD here, was kindly donated by Dr Iino and Hirose *et al.* (1999).

### Cell culture

SH-SY5Y cells were cultured in Dulbecco’s modified Eagles’ medium (Nacali tesque, Kyoto, Japan) and Dulbecco’s modified Eagles’ medium/Ham’s F-12 medium (1 : 1) (GIBCO, Grand Island, NY, USA) at 37°C in humidified atmosphere containing 5% CO_2_. The media contained 25 mmol/L glucose, and were buffered with 44 mmol/L NaHCO_3_ and supplemented with 10% fetal bovine serum (Sigma), 1% GlutaMAX^TM^-I (Invitrogen Corp., Carlsbad, CA, USA), penicillin (100 units/mL) and streptomycin (100 μg/mL) (Invitrogen Corp.).

### Constructs of plasmids encoding GFP-fused εPKC and mutants

The plasmid encoding εPKC having GFP at its C terminus [full length εPKC (εFL)–GFP] was described previously (Shirai *et al.* 1998). A cDNA fragments encoding εRD with Bgl II site was produced by a PCR with cDNA for rat εPKC as the template. The sense primer was 5-TTAGATCTACCATGGTAGTGTTCAATGGCC-3, and the anti-sense was 5-GAAGATCTTCCTCGGTTGTCAAATGAC-3. The PCR product was subcloned into pTB701-GFP (described as BS340 in Shirai *et al.* 1998) for the expression in mammalian cells and into pGEX-4T-1 (Amersham Pharmacia Biotech, Buckinghamshire, UK) for bacterial expression of a glutathione *S*-transferase (GST)-fusion protein, respectively. The plasmids encoding mutants lacking variable region 1 (ΔV1), pseudo-substrate (ΔPS) region, C1, C1A, C1B domains, and V3 region were made using the amino terminus-deleted and domain-deleted mutants of εPKC described by Kashiwagi *et al.* (2002) as the template and primers described above. Similarly, PCR was performed using following primers to make mutants lacking some of V3 regions, and subcloned into Bgl II site of BS340. Anti-sense primers were 5-TTAGATCTTTCCTGGTCACAAGGGGA-3 for RRKK mutant II, 5-GAAGATCTTGACTTGGATCGGTCGTCTTC-3 for RRKK mutant, and 5-TTAGATCTTGGCCACTGTTGAT-3 for ΔRRKK, respectively. Furthermore, a site directed mutagenesis was performed according to manufacture’s recommended protocol with ExSite PCR-based site directed mutagenesis kit (Stratagene, La Jolla, CA, USA) to make the putative actin-binding site-Δ (ΔpABS) mutant. Rat εPKC cDNA was used as a template and the primers used were 5′-ATCAACAACATCCGGAAGGCC-3′ and 5′-ATCTTCCTGGTCACAAGGGGA-3′. All PCR products were verified by sequencing.

### Constructs of plasmids encoding a chimera of PH domain of PLCδ and the V3 region of εPKC

A cDNA fragment encoding the V3 region of rat εPKC with Xho I sites were produced by PCR using the sense primer, 5-TTTCTAGAGGGGTGGACGCCAGAGGAATT-3, and the anti-sense primer, 5-CCGGGCCCTTATCCTCGGTTGTCAAATGA-3. A cDNA encoding PH domain was obtained by digestion of GFP–PHD by with Bam HI and Xho I. The PCR product of the εPKC V3 region and the cDNA encoding PHD were subcloned into pEGFPC1 (Clontech, Palo Alto, CA, USA). The PCR product was verified by its sequencing.

### Transfection and confocal microscopy

Cells (2.0 × 10^6^ cells/dish) on glass-bottom dish (MatTek Corp., Ashland, MA, USA) were transfected using 6 μL of FuGENE^TM^6 transfection reagent (Roche Molecular Biochemicals) and 2 μg of DNA according to the manufacturer’s protocol. Transfected cells were cultured at 37°C for about 24 h before use. The fluorescence of GFP was observed under confocal laser scanning fluorescent microscopy (Carl Zeiss, Jena, Germany). The GFP-fluorescence was monitored at 488 nm argon laser excitation with 515 nm long pass barrier filter.

### Evaluation of neurite induction

Twenty-four hours after the transfection, at least 100 cells expressing the GFP-fused εPKC or mutants were observed under confocal laser microscopy and the cells with neurites longer than the length of two cell bodies were counted. Three independent experiments were performed and data (mean + SEM) are presented as percentage of the cells having long neurites among the 100 cells expressing GFP-fused εPKC or mutants

### Recombinant protein expression and purification

BL21 (DE3) pLys cells were transformed with the plasmids for GST–εRD and control vector according to the manufacture’s instruction (New England Biolabs, Beverly, MA, USA), and the expression of recombinant proteins was induced by 0.1 mmol/L isopropyl-1-thio-β-d-galactoside at 25°C for 4 h. The cells were then harvested and lysed in column buffer [20 mmol/L Tris–HCl (pH 7.0), 1 mmol/L EDTA, 1 mmol/L dithiothreitol, 5 mmol/L MgCl_2_, 250 mmol/L sucrose, 1% triton-X 100, 20 μg/mL leupeptin, and 1 mmol/L phenylmethylsulfonyl fluoride] with handy sonic (Tomy Seiko Co., Ltd, Tokyo, Japan). After ultra-centrifugation at 14 000 *g* for 30 min, the proteins were purified using Glutathione-Sepharose 4B (Amersham Pharmacia Biotech) according to the manufacture’s instruction.

### Protein-lipid overlay assay

Various amount of PIP_2_ (2–500 pmol) were spotted on Hybond-C extra membrane (Amersham Biosciences). After dried up, the membrane was blocked with 3% fatty acid-free bovine serum albumin in Tris buffered saline with Tween 20 (TBST; 50 mmol/L Tris–HCl, 150 mmol/L NaCl, and 0.1% Tween 20, pH 7.5) for 1 h at 25°C, and then incubated with 20 nmol/L GST–RDεPKC or GST for 3 h at 25°C in the blocking solution. The membrane was washed five times for 1 h (each time) in TBST buffer and then incubated for 1 h with 1/1000 dilution of anti-GST polyclonal antibody (Sigma-Aldrich) in the blocking solution. After washing with TBST, the membrane was immersed in the blocking solution with anti-rabbit horseradish peroxidase conjugate (1/5000 dilution) (Jackson ImmunoResearch Laboratories, West Grove, PA, USA) for 1 h. After washing, the protein bound to the lipid was detected by enhanced chemiluminescence (Amersham Biosciences).

In the case of protein-lipid overlay (PLO) assay using GFP fusion proteins, we used lysate of COS-7 cells, which were transfected with various plasmids encoding εPKC and the mutants. The amount of the fusion proteins to be applied to the PLO assay was adjusted by western blot using GFP antibody.

## Results

### Effect of over-expression of εPKC on neurite extension

**Fig. 1:**
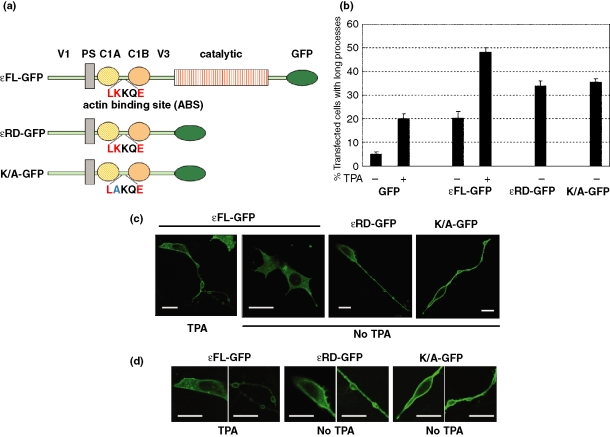
Induction of neurite outgrowth by over-expression of protein kinase C-ε (εPKC) and mutants. (a) Schematic illustration of full length εPKC (εFL), regulatory domain of εPKC (εRD) and regulatory domain of K/A mutant. Lysine in the actin-binding site of the K/A mutant was substituted by alanine. Consensus amino acids in the actin-binding motif were written in red. (b) Statistic analysis on the neurite induction ability of εPKC and the mutants. Twenty-four hours after the transfection, the cells with neurites longer than the length of two cell bodies were counted as described in Materials and methods. Three independent experiments were performed and the data (mean + SEM) are presented as percentage of the cells having long neurites among the cells expressing green fluorescent protein (GFP)-fused εPKC or mutants. (c) Typical neurites induced by GFP-tagged εFL, regulatory domain of εPKC (εRD), and KA mutant in the presence or absence of 12-*ο*-tetradecanoylphorbol-13-acetate (TPA) and their localizations. Plasmids encoding GFP-tagged εFL, εRD, and KA mutant were transfected into SHSY-5Y cells by lipofection and images were taken after 24 h after the transfection. Bars are 20 μm. (d) Magnified images of SH-SY5Y cells expressing GFP-tagged εFL, εRD, or KA mutant. The images shown in (c) are magnified to clearly show the plasma membrane localization of GFP–εFL in the presence of TPA, and of εRD or KA mutant without any stimulation. Bars are 20 μm.

First, we tried to confirm the neurite induction by over-expression of εPKC in SH-SY5Y cells. Approximately 20% of the cells expressing εFL–GFP had long neurites, while only 5% of control cells expressing GFP showed long processes (Fig. 1b). εFL–GFP was expressed mainly in the cytoplasm under the normal conditions but TPA treatment induced translocation of εFL–GFP to the plasma membrane (Fig. 1c), resulting in the neurite extension; about 50% of the εFL–GFP cells possessed long neurites in the presence of TPA (Fig. 1b). Typical long neurite induced by εPKC had one or two neurites with varicose structures (Fig. 1c).

By contrast, εRD–GFP showed membrane localization and strong neurite-induction activity even in the absence of TPA (Fig. 1b and c); 35% of the εRD cells had the typical long neurite. These results are basically consistent with previous findings by Larsson’s group.

To investigate the importance of actin-binding site between C1A and C1B in the neurite induction by εRD, we made a K/A mutant, in which lysine in the consensus sequence of the actin-binding site was substituted by alanine. RD of the K/A mutant showed both strong neurite induction activity and membrane localization as shown in Fig. 1b and c; about 35% of the cells expressing RD of the K/A mutant possessed long neurites. These results suggested that, except for the actin-binding site, unknown important factor is involved in the εRD-induced neurite induction

### PIP_2_ binding of εPKC

**Fig. 2:**
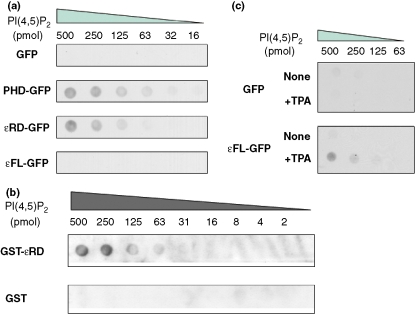
Phosphatidylinositol 4,5-bisphosphate (PIP_2_) binding of protein kinase C-ε (εPKC). (a) Binding of regulatory domain of εPKC (εRD) to PIP_2_. Various amount of PIP_2_ were spotted on the membrane and protein-lipid overlay (PLO) assay was performed using lysates from COS-7 cells expressing εRD–green fluorescent protein (GFP) or full length εPKC (εFL)–GFP. GFP and GFP–pleckstrin homology domain (PHD) of PLCδ were used as negative and positive control, respectively. (b) Direct binding of εRD to PIP_2_. Purified GST–εRD (10 nmol/L) was used for PLO assay to show direct binding. Same concentration of purified glutathione *S*-transferase (GST) was used as negative control. (c) 12-*ο*-tetradecanoylphorbol-13-acetate (TPA)-dependent PIP_2_ binding of full length εPKC (εFL). PLO assay was performed using lysates from COS-7 cells expressing εFL–GFP or GFP in the presence and absence of 200 nmol/L TPA.

We, then, focused on PIP_2_ as a possible candidate for the important regulator of the εRD-induced neurite induction as described above. To examine whether εPKC can bind to PIP_2_, PLO assay was employed using GFP-fusion proteins from COS-7 cell lysate_._εRD–GFP clearly binds to PIP_2_ as well as PH domain of phospholipase Cδ, which is well known to specifically bind to PIP_2_ (Fig. 2a). To confirm that εRD–GFP directly binds to PIP_2_, purified recombinant protein of GST–tagged εRD (GST–εRD) was used in the PLO assay. Similarly to the GFP-fusion protein form COS-7 cell lysate, the purified protein bound to PIP_2_ in a dose-dependent manner (Fig. 2b), indicating εRD can directly bind to PIP_2_ without any additional factors. By contrast, εFL did not show PIP_2_ binding under the same conditions. We, then, performed PLO assay in the presence of TPA because TPA induced the membrane localization of εPKC and neurite outgrowth as shown in Fig. 1. As expected, the TPA treatment enabled εFL to bind to PIP_2_ (Fig. 2c).

**Fig. 3:**
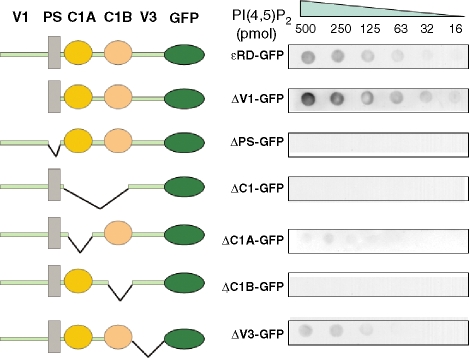
Schematic illustration and phosphatidylinositol 4,5-bisphosphate (PIP_2_) binding ability of the domain-deleted mutant of protein kinase C-ε (εPKC). Protein-lipid overlay (PLO) assay was performed using lysates from COS-7 cells expressing green fluorescent protein (GFP)-fused regulatory domain of εPKC (εRD), and deletion mutants of variable region 1 (ΔV1), pseudo-substrate (ΔPS), common region 1-Δ (ΔC1), first half of C1 domain (ΔC1A), second half of C1 domain (ΔC1B), and ΔV3.

To identify the responsible region for the PIP_2_ binding, we made a series of mutants and investigated their PIP_2_ binding (Fig. 3). A mutant lacking V1 region from RD (ΔV1–GFP) showed stronger PIP_2_ binding than εRD–GFP, while deletion of V3 region weakened the PIP_2_ binding. Deletion of either PS or C1 domain completely abolished the PIP_2_ binding, indicating there is PIP_2_ binding region(s) in the PS and C1 domains. Furthermore, to investigate which domain, C1A or C1B, is critical for the PIP_2_ binding, we made C1A or C1B-deleted mutants. ΔC1A–GFP very faintly bound to PIP_2_, while ΔC1B–GFP lost the PIP_2_ binding. These results suggest that PS and C1 regions, relatively C1B than C1A, are important for the PIP_2_ binding.

### Importance of PIP_2_ binding for the neurite induction

**Fig. 4:**
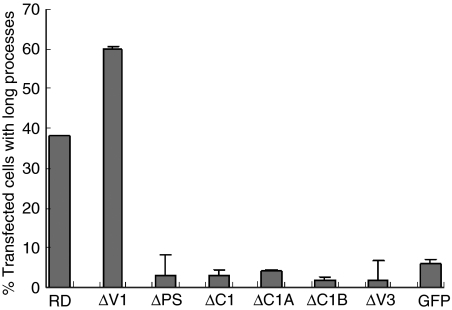
Neurite induction ability of the domain-deleted mutant of protein kinase C-ε (εPKC). Twenty-four hours after the transfection of respective plasmids, the cells with neurites longer than the length of two cell bodies were counted. Three independent experiments were performed and at least 100 cells were counted at every experiment. Data (mean + SEM) are presented as percentage.

**Fig. 5:**
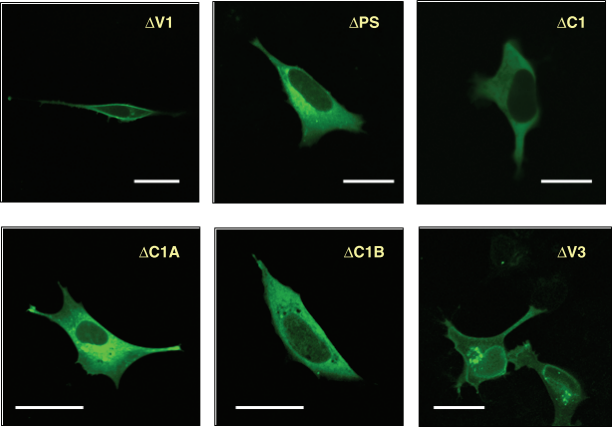
Localization of the domain-deleted mutant of protein kinase C-ε (εPKC). Plasmids encoding green fluorescent protein (GFP)-tagged mutants deleted variable region 1 (ΔV1), pseudo-substrate (ΔPS), common region 1-Δ (ΔC1), first half of C1 domain (ΔC1A), second half of C1 domain (ΔC1B), and ΔV3 were transfected into SHSY-5Y cells by lipofection and images were taken after 24 h after the transfection. Bars are 20 μm.

Next, the effects of over-expression of the deletion mutants on neurite induction were examined. ΔV1–GFP showed stronger neurite-induction activity than εRD–GFP; 60% of cells over-expressing of ΔV1–GFP possessed long neurite (Fig. 4). On the other hand, ΔPS, ΔC1, ΔC1A, ΔC1B–, and ΔV3–GFP lost ability to induce long neurite (Fig. 4). Localization of the mutants was also investigated. ΔV1–GFP showed very clear membrane localization, while ΔPS, ΔC1, ΔC1A, and ΔC1B–GFP were cytosolic. ΔV3–GFP was localized on the nuclear membrane with perinuclear accumulation, in addition to weak plasma membrane localization (Fig. 5).

Table 1 summarizes the results of localization, PIP_2_ binding activity and neurite induction activity, indicating that the mutants without PIP_2_ binding activity clearly lost membrane location and neurite induction. On the other hand, the mutant having stronger PIP_2_ binding activity showed enhanced neurite induction ability. These results demonstrated that PIP_2_ binding is indispensable and important for the membrane localization and neurite induction of εPKC. In addition, as seen in the result of ΔV3–GFP, moderate PIP_2_ binding was not enough for the neurite induction, suggesting that strong PIP_2_ binding and/or V3 region are necessary.

**Table 1:** Correlation between the phosphatidylinositol 4,5-bisphosphate (PIP_2_) binding, the plasma membrane localization, and the neurite extension of protein kinase C-ε (εPKC)

	PIP_2_ binding	Plasma membrane localization	Neurite extension
εPKC without TPA	No	No	20%
εPKC without TPA	++	Yes	50%
Wild RD	++	Yes	35%
RDK/A	++	Yes	35%
RD ΔV1	+++	Yes	60%
RD ΔPS	No	No	6%
RD ΔC1	No	No	5%
RD ΔC1A	Very faint	No	6%
RD ΔC1B	No	No	2%
RD ΔV3	+	Weak	5%

+++ and + represents stronger and moderate PIP_2_ binding, respectively. TPA, 12-*ο*-tetradecanoylphorbol-13-acetate; V1, variable regions 1 and 3; PS, pseudo-substrate; C1, common region 1; C1A, first half of C1 domain; C1B, second half of C1 domain; RD, regulatory domain.

**Fig. 6:**
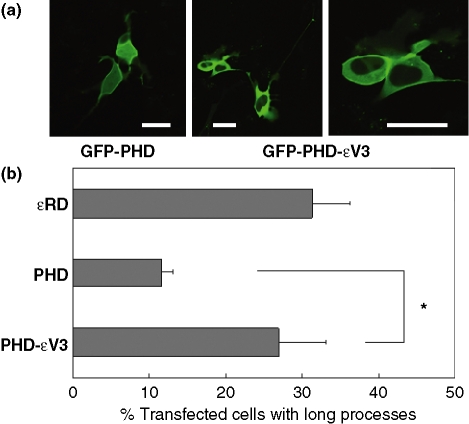
Effect of pleckstrin homology domain (PHD) of phospholipase C-δ (PLCδ) and the variable region 3 (V3) of protein kinase C-ε (εPKC) on neurite induction. (a) Localization of green fluorescent protein (GFP)–PHD and GFP–PHDεV3 in SY-SY5Y cells. Bars are 20 μm. (b) Statistical analysis on the neurite induction ability of GFP–PHD and GFP–PHDεV3. Twenty-four hours after the transfection, the cells with neurites longer than the length of two cell bodies were counted. Three independent experiments were performed and at least 100 cells were counted at every experiment. Data (mean + SEM) are presented as percentage. *p < 0.05.

To confirm the importance of the PIP_2_ binding and V3 region for the neurite induction, we made a chimera of PH domain of PLCδ and V3 region of εPKC (PHD–εV3) and compared its neurite induction ability with that of PHD alone. Approximately 10% of the cells expressing PHD possessed long neurites, while 28% of the cells having PHD–εV3 showed comparable neurite induction ability to the εRD, although both proteins showed membrane localization (Fig. 6). These results indicate PIP_2_ binding and V3 region are sufficient for the neurite induction.

### Identification of subregion in the V3 region important for the neurite induction

**Fig. 7:**
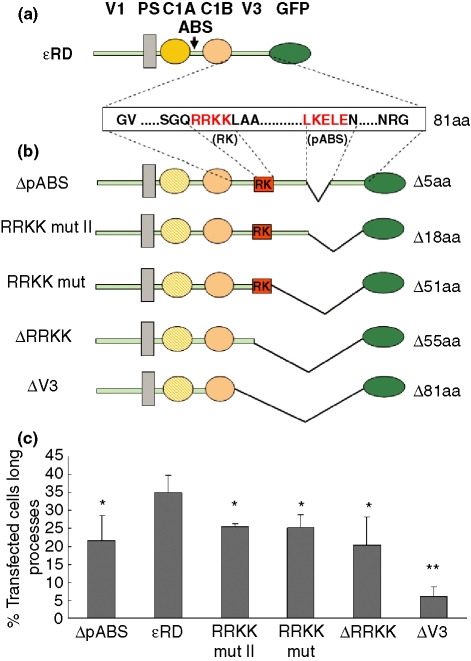
Neurite induction ability of the protein kinase C-ε (εPKC) mutants lacking some of variable region 3 (V3). (a) V3 region of rat εPKC consists of 81 amino acids including a cluster of basic amino acids RRKK and putative actin-binding motif, LKELE. (b) Schematic illustration of the mutants. RK and putative actin-binding site (pABS) represent the cluster of basic amino acids and the putative actin-binding site in the V3 region, respectively. The numbers at the right side indicates the numbers of deleted amino acids from the V3 region. (c) Statistical analysis on the neurite induction ability of regulatory domain of εPKC (εRD) and the mutants. Twenty-four hours after the transfection, at least 100 cells expressing respective mutants were observed and the ratios of the cells with neurite longer than the length of two cell bodies to all observed cells were represented as percentage. Data are presented as mean ± SEM in three independent experiments. *Indicates the difference between εRD and the respective mutants was significant (*p*< 0.05). **Indicates the difference between ΔV3 and ΔRRKK was significant (*p* < 0.05).

**Fig. 8:**
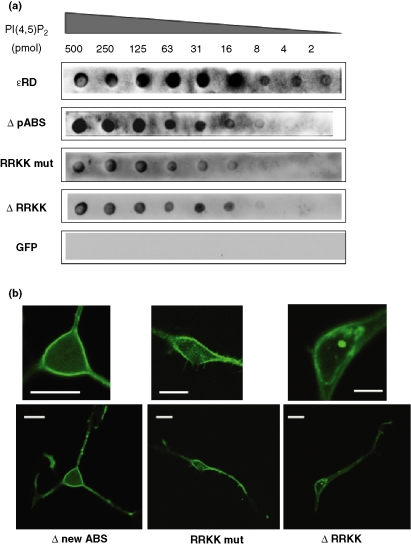
Phosphatidylinositol 4,5-bisphosphate (PIP_2_) binding ability and localization of the protein kinase C-ε (εPKC) mutants lacking some of variable region 3 (V3). (a) Protein-lipid overlay (PLO) assay was performed using lysates from COS-7 cells expressing green fluorescent protein (GFP)-fused Δputative actin-binding site (pABS), RRKK mutant, or ΔRRKK. Regulatory domain of εPKC (εRD) and GFP were used as positive and negative control, respectively. (b) Plasmids encoding GFP-tagged ΔpABS, RRKK mutant, or ΔRRKK were transfected into SHSY-5Y cells by lipofection and images were taken after 24 h after the transfection. Upper images are magnified ones. Upper panels are magnified images. Bars are 20 μm.

To identify subregion(s) of the V3 region important for the neurite induction, we focused on two regions; that are a cluster of basic amino acids corresponding to amino acids from 319 arginine to 322 lysine (RRKK) and a putative ABS corresponding to amino acids from 356 leucine to 360 glutamic acid (pABS), which has consensus actin-binding motif LKXXEX (Fig. 7a). We made a series of the mutants as shown in Fig. 7b and compared their neurite induction ability. Both RRKK and RRKK II mutants showed about 30% less neurite induction ability compared with the εRD; about 25% of the cells expressing the RRKK and RRKK II mutants had typical neurites. The PIP_2_ binding of RRKK was slightly weaker than εRD but were strongly enough (Fig. 8a). The PIP_2_ binding of RRKK II mutant was similar to that of RRKK mutant This suggested that pABS is somehow important for the neurite induction and PIP_2_ binding, because both RRKK and RRKK II mutants lack pABS. Consistently with this, the ability of the pABS deletion mutant to induce the neurite and its PIP_2_ binding was lower than that of εRD and similar to those of RRKK and RRKK II (Figs 7 and 8). On the other hand, deletion of the basic amino acid cluster form RRKK (ΔRRKK) did not significantly affect on the neurite induction ability and the PIP_2_ binding (Fig. 7). However, when further 16 amino acids were deleted from ΔRRKK mutant lost the neurite induction ability. The localization of these mutants was also investigated. As shown in Fig. 8b, ΔpABS and RRKK mutant were localized on the plasma membrane. The localization of RRKK II mutant was similar to that of RRKK mutant (data not shown). Some cells expressing ΔRRKK showed the nuclear membrane localization in addition to the plasma membrane localization like ΔV3 mutant (Fig. 8b), suggesting the basic amino acids are important for the plasma membrane localization.

These results revealed that the first 16 amino acids of the V3 region are important for the neurite induction and confirmed the importance of the PIP_2_ binding on the neurite induction.

## Discussion

This is a first report to show that εPKC directly binds to PIP_2_. There are two types of interaction between PIP_2_ and proteins. One is the case that some proteins interact with the phosphoinositide through distinct domains including PH domain, FYVE, and ENTH, etc (DiNitto *et al.* 2003). The other case is that partner proteins bind to the lipid through an electrostatic mechanism involving basic amino acids. Generally, it is considered that the former interaction is more specific than the latter’s, although there are varieties of the specificity even in the case of the interaction between the distinct lipid-binding domains and PIP_2_ (DiNitto *et al.* 2003). Looking at the responsible region of εPKC for the PIP_2_ binding, specific motives for phosphoinositides like PH or five domains are not found, suggesting that electrostatic mechanism of basic amino acids are involved in the PIP_2_ binding of εPKC. Indeed, the PS and C1 regions of εPKC contain many basic amino acids and mutations on some of them reduced the PIP_2_ binding (data not shown). Especially, a number of basic amino acids in the PS region of εPKC are prominent among PKCs. Alternatively, higher structure of the C1 domain may be important as its structure is critical for DAG binding (Kazanietz *et al.* 1995; Xu *et al.* 1997). NMR and crystallography of the PS and C1 with PIP_2_ would be informative to understand precise mechanism of the PIP_2_ interaction. In the meantime, the characteristics of the PIP_2_ binding sites suggest that εPKC may bind to another phospholipids including PIP_3_. Interestingly, the regulations of εPKC by PIP_3_ have been reported (Toker *et al.* 1994; Moriya *et al.* 1996).

We also showed that the PIP_2_ binding ability of εPKC and mutants well correlated to their neurite induction abilities. For example, εFL induced neurites and bound to PIP_2_ in the presence of TPA, while any stimulation was not necessary for neurite induction and the PIP_2_ binding of εRD. In addition, the PIP_2_ binding abilities of the domain-deleted mutants of εPKC were parallel to their neurite induction abilities (Table 1 and Fig. 8). These results indicate that the PIP_2_ binding is important for the neurite induction. However, the PIP_2_ binding was not sufficient for the neurite outgrowth because PH domain of PLCδ did not induce the neurite outgrowth in SHSY-5Y cells. For the neurite induction, the V3 region of the εPKC was additionally necessary. It is evident that the V3 region alone was not enough either, because the mutant having the V3 regions, for example, ΔPS and ΔC1 lost the ability to induce neurite outgrowth. These results clearly show that both the PIP_2_ binding and εPKC V3 region are necessary and sufficient for the neurite induction. Importantly, the results using PHD–εV3 also proved that, at least, PIP_2_ is responsible lipid for the neurite induction even if εPKC could bind to another phospholipids, based on that PH domain of PLCδ specifically binds to PIP_2_.

In the V3 region of rat εPKC, we found pABS and it seemed to be somehow important for the neurite induction ability because deletion of the pABS reduced the ability about 20%. We, therefore, investigated whether the pABS binds to actin by ELISA and Biacore 3000 (GE Healthcare, Buckinghamshire, UK) using purified GST-fusion protein of the V3 region (GST–εV3) and pABS deleted one (GST–εV3ΔpABS). Unfortunately, however, we could not conclude that the V3 region binds to actin through the pABS because both GST–εV3 and GST–εV3ΔpABS showed tendency to bind to actin and the interaction was not so significant (data not shown). In addition, the pABS is not conserved in human εPKC although it is seen in mouse εPKC; the corresponding sequence of human is insufficient as actin-binding motif. In spite of this, human εPKC also induces the neurite. These facts suggest that pABS does not functions as ABS. Rather, the first 16 amino acids in the V3 region are important for the neurite induction of εRD. The importance of these amino acids for neurite induction is supported by Ling *et al.* (2005); they showed first 20 amino acids of V3 region are critical for the neurite induction ability of human εPKC.

Furthermore, plasma membrane localization appears to be important for the neurite induction for εPKC because all mutants which induced neurite outgrowth were localized on the plasma membrane, consistent with previous results (Zeidman *et al.* 1999, 2002). On the other hand, ΔV3 mutants, which lost the neurite induction activity, showed nuclear and Golgi membrane localization in addition to weak plasma membrane localization, suggesting V3 region and PIP_2_ binding may contribute to the membrane localization. Indeed, importance of a cluster of basic amino acids, RRKK, in the V3 region was shown (Fig. 8). However, the plasma membrane localization and the PIP_2_ binding were not sufficient for the neurite induction because GFP–PHD did not induce neurite although it localizes on the plasma membrane. These findings suggest recruitment of εPKC to specific site on the plasma membrane may be important for the neurite induction.

How do the PIP_2_ binding and the V3 region, especially the first 16 amino acids, participate in the neurite outgrowth? What we can do here is only speculation. One plausible function of the PIP_2_ binding of εPKC is to regulate the amount of PIP_2_ at local sites of the membrane. This may influence the function of actin-binding proteins, resulting in actin rearrangement and neurite induction. Other possibility is that the PIP_2_ binding of εPKC contributes to the inhibition of the ROCK-Rho A path way because Larsson’s group reported that Rho A and ROCK are involved in the εRD-induced neurite outgrowth (Trollér *et al.*, 2004). Indeed, PIP_2_ activates Rho A by regulating open state of RhoA/RhoGDI complex (Faure *et al.*, 1999). The PIP_2_ binding of εPKC may affect on this state by reducing amount of free PIP_2_ at specific site. On hand, the V3 region may bind to some proteins, the regulating RhoA/ROCK pathway and contributing to the recruitment of εPKC to appropriate site. A GTPase-activating protein for the Rho family, p190RhoGAP, is one interesting candidate to bind to the V3 because it binds to εPKC (Ling *et al.* 2005) and is relating to the regulation of Rho A. Intriguingly, the accumulation of p190 RhoGAP in lipid rafts regulates Rho activity, affecting in cytoskeletal structure (Mammoto *et al.* 2006). As discussed, the mechanism of εPKC-induced neurite induction is still puzzling but the PIP_2_ binding would be one piece to solve the puzzle, in addition to several proteins including actin, actin-binding proteins, Rho A, and Rho-regulating proteins as reported by Larsson group.

In conclusion, εPKC binds to PIP_2_ through the PS and C1 regions, contributing its neurite induction ability cooperating with the V3 region. The PIP_2_ binding gives new insight to understand mechanisms of εPKC functions including the neurite induction.

## Abbreviations used

ABS: actin-binding site
C1: common region 1
C1A: first half of C1 domain
C1B: second half of C1 domain
DAG: diacylglycerol
εFL: full length εPKC
GAP: GTPase-activating protein
GFP: green fluorescent protein
GST: glutathione *S*-transferase
PHD: pleckstrin homology domain
PIP_2_: phosphatidylinositol 4,5-bisphosphate
PKC: protein kinase C
PLC: phospholipase C
PLO: protein-lipid overlay
PS: pseudo substrate
ΔpABS: putative actin-binding site-Δ
RD: regulatory domain
TBST: Tris buffered saline with Tween 20
TPA: 12-*ο*-tetradecanoylphorbol-13-acetate
V1 and V3: variable regions 1 and 3
εRD: regulatory domain of εPKC
